# Synthesis and Aqueous Solution Properties of an Amino Bisphosphonate Methacrylate Homopolymer via RAFT Polymerization

**DOI:** 10.3390/polym10070711

**Published:** 2018-06-27

**Authors:** Panagiotis G. Falireas, Claire Negrell, Ghislain David

**Affiliations:** Institut Charles Gerhardt—UMR-CNRS 5253, Ingénierie et Architectures Macromoléculaires, ENSCM, 8 rue de l’Ecole normale, CEDEX 5, 34296 Montpellier, France; Panagiotis.Falireas@enscm.fr (P.G.F.); claire.negrell@enscm.fr (C.N.)

**Keywords:** amino bisphosphonate, RAFT polymerization, solvent polarity, hydrolysis, amino bisphosphonic acid, pH-responsive polymers

## Abstract

The present contribution reports on the synthesis via reversible addition-fragmentation chain transfer (RAFT) polymerization of a methacrylate derivative bearing an aminobisphosponate moiety as a pendant group, namely, ethyl *N*,*N*-tetramethylbis(phosphonate)-bis(methylene) amine methacrylate (MAC_2_NP_2_). The polymerization was performed by the use of cyanoisopropyl dithiobenzoate as chain transfer agent at 70 °C in various solvents with different polarities including *N*,*N*-dimethylformamide, acetonitrile, tetrahydrofuran, and in bulk. Best results were obtained in *N*,*N*-dimethylformamide where higher conversions and polymerization rates were noticed. The successful hydrolysis of the phosphonate ester groups was performed using bromotrimethylsilane with excellent yields leading to the formation of highly water soluble and pH-responsive polymers. Finally, a preliminary solution behavior study was carried out by investigating the aqueous solution properties of synthesized amino bisphosphonate methacrylate homopolymers and their phosphonic acid analogs via potentiometric titration and zeta potential measurements.

## 1. Introduction

It is well known that phosphorus-based polymers represent a unique class of materials, which exhibit exceptional and unique properties, thus, they have gained wide appeal and interest for scientific and industrial research within the last decades. Particularly, they have been employed for a wide range of technological applications such as dental adhesives [1], proton exchange membrane [2,3], flame retardant materials [4,5], bind metal ions [6,7], and in the biomedical field as drug carries or for tissue engineering as they exhibit excellent biodegradability and biocompability [8,9,10]. In general terms, phosphonated monomers are notably difficult to polymerize to full conversion [11,12], therefore, the most approachable method towards the synthesis of polymers bearing phosphorus functionalities along the backbone is by post-functionalization of polymers with phosphorus-containing groups [13,14,15]. However, during the past two decades, the synthesis of these niche polymers has been facilitated by the tremendous progress of pseudo-living radical polymerization techniques, which allow engineering well-defined polymers with tunable compositions, structures, and properties. Atom transfer radical polymerization (ATRP) and RAFT have been widely used for the controlled polymerization of monomers carrying phosphates [16,17,18], phosphinic acids [19,20], phosphonic acids [21], and phosphonates [12,22,23,24,25,26] as pendant groups. The latter polymerization method has been one of the most effective and versatile methods which allows the control synthesis of a wide variety of monomers and is also compatible with a wide variety of reaction conditions. A great advantage of RAFT is the use of chain transfer agent (CTA) which can replace the use of potentially dangerous or toxic transition metal catalysts. Different types of CTAs have been studied such as dithioesters, dithiocarbamates, trithiocarbonates, and xanthates. Among them, dithioesters/dithiobenzoates and trithiocarbonate have been vastly used on the controlled polymerization of (meth)acrylate, (meth)acrylamide, and styryl derivatives due to the high compatibility with the aforementioned monomers [27]. Moreover, RAFT polymerization leads to the synthesis of well-defined polymers with narrow polydispersities, predetermined molecular weights, functionalized end groups, and polymers with complex architectures such as linear block copolymers, comb-like, brush polymers, star, dendrimers and cross-linked networks. Additionally, RAFT has successfully combined with the step-growth polymerization resulting in well-defined high molar mass segmented copolymers [28]. Finally, in the recent years, RAFT polymerization has a lot of emerging industrial potential as it has been used for the synthesis of narrowly dispersed polymers via micro-flow technology [29].

In the same context, amino bisphosphonates and bisphosphonates are a special class of phosphorus-containing compounds and can find potential applications in many fields. In the area of medicine, these materials are widely used due to their low toxicity and they are considered as primary agents in the pharmacological arsenal for treatment against osteoporosis due to their ability of binding to the mineral phase of bone and inhibit the activity of osteoclasts [30]. Furthermore, the corresponding bisphosphonic acids can serve as efficient tridentate metal chelators or metalloid ions, chelating through both phosphonate groups and the lone pair on the nitrogen atom [31,32]. Additionally, they can be used very efficiently as scale inhibitors in the oil industry [33]. Finally, polymers bearing amino bisphosphonates groups showed outstanding properties when used as inhibitors for anti-corrosive coatings or used as flamed retardants, enhancing char residues of polymeric materials [34,35,36].

Despite the fact that bisphosphonates, amino bisphosphonates, and their phosphonic acid derivatives exhibit interesting properties, academic reports based on the direct synthesis of polymers containing them as pendant groups are scarce. Sumerlin et al. [37] reported the synthesis of polyacrylamides containing pendant amino bisphosphonate groups via RAFT polymerization. In a first step, well-defined polyacrylamides were synthesized followed by post-polymerization functionalization with amino bisphosphonate moieties by using the Kabachnik–Fields reaction, with a functionalization degree of 75% being achieved. As far we know, the first reference for the synthesis and direct polymerization of a phosphonic methacrylate incorporating an amino bisphosphonic group was reported by our group. Particularly, by using an elegant synthetic method the synthesis of a series of (*N*,*N*) bismethylene diphosphonate methacrylates was achieved, varying the spacer of the methylene linking groups, namely *N*,*N*-tetramethyl-bis(phosphonate)-bis(methylene) amine methacrylates (MAC*_n_*NP_2_/MMA (2 ≤ *n* ≤ 11)). The synthesized monomers were copolymerized with methyl methacrylate (MMA) via conventional polymerization yielding statistical copolymers [38]. However, to the best of our knowledge, controlled radical polymerization of monomers bearing amino bisphosphonate moieties was surprisingly never reported in the academic literature.

Towards this direction, the present study reports on the synthesis and characterization of an amino bisphosphonate methacrylate with a C_2_ spacer and polymerization thereof via RAFT. The kinetic behavior and the evolution of number average molar mass were studied when solvents with different polarities were used and also in bulk conditions. Subsequent conversion of phosphonate esters to their phosphonic acid analogs groups was carried out yielding polymer with pH-responsive properties where their solution properties were studied in a salt-free aqueous solution as a function of pH by potentiometric titration and zeta potential measurements. The synthesis of these materials could create new perspectives for the development of systems for use in a wide range of applications.

## 2. Materials and Methods

### 2.1. Materials

Ethanolamine ≥98%, paraformaldehyde 95%, dimethyl phosphite 98%, methacryloyl chloride 97%, triethylamine (TEA) ≥99%, 2-cyano-2-propyl-benzodithioate (CPDB) ≥97%, bromotrimethylsilane 97% (TMSBr), *N*,*N*-dimethylformamide (DMF), tetrahydrofuran (THF), dichloromethane (DCM), acetonitrile (CH_3_CN), 1,4-dioxane, methanol (MEOH), *tert*-butyl methyl ether were purchased by Sigma Aldrich and were used as received. 2,2′-azobis(2-methylpropionitrile) 98% (AIBN) was recrystallized twice from methanol. Any glassware was cleaned in a KOH/isopropanol bath and dried under vacuum prior use.

### 2.2. Synthesis of MAC_2_NP_2_ Monomer

The synthesis of the monomer was carried-out by a two-step reaction process according to previously reported work [38]. The successful synthesis of the MAC_2_NP_2_ monomer was confirmed by proton and phosphorus nuclear magnetic resonance (^1^H NMR and ^31^P NMR) spectroscopy (see Figures S1 and S2, Supporting Information). ^1^H NMR (400 MHz, CDCl_3_) δ: 1.86 (s, 3H), 3.05–3.08 (t, 2H), 3.16–3.18 (d, 4H), 3.68–3.71 (d, 12H), 4.17–4.20 (t, 2H), 5.50 (s, 1H), 6.03 (s, 1H). ^31^P NMR (162 MHz, CDCl_3_) δ: 26.42–26.65 (quintuplet).

### 2.3. Synthesis of PMAC_2_NP_2_ via RAFT Polymerization

A typical procedure for the synthesis of the MAC_2_NP_2_ homopolymer by RAFT is described below. MAC_2_NP_2_ (2 g, 5.36 mmol), CPDB (38 mg, 0.17 mmol), and AIBN (9.4 mg, 0.06 mmol) were added in a round bottom Schlenk flask equipped with a stir bar under a nitrogen flow followed by the addition of CH_3_CN (10 mL). The flask was sealed with a rubber septum, and the reaction mixture was stirred for 20 min at RT after being degassed and filled with nitrogen. Subsequently, the mixture was degassed by three freeze-pump-thaw cycles and the reaction was allowed to proceed at 70 °C in a thermostated oil bath for an appropriate time. Aliquots (0.4 mL) were taken at an interval of 1 h under a nitrogen atmosphere using a nitrogen-purged syringe and were immediately frozen to quench the polymerization. Finally, the polymerization was terminated by immersing the flask in liquid nitrogen and opened to air. The purified product was obtained after two repeated precipitations in cold *tert*-butyl ether, was dried under vacuum overnight, and was characterized by ^1^H NMR, ^31^P NMR, spectroscopy and gel permeation chromatography. (See Figures S3 and S4, Supporting Information). ^1^H NMR (400 MHz, CDCl_3_) δ: 0.83–1.00 d, (3H), 1.80 (s, 2H), 3.05 (s, 2H), 3.22 (s, 4H), 3.75–3.77 (d, 12H), 4.04 (s, 2H). ^31^P NMR (162 MHz, CDCl_3_) δ: 26.55 (s).

### 2.4. Hydrolysis of the Phosphonated Ester Groups of PMAC_2_NP_2_

After the successful synthesis of the homopolymer in DMF, the phosphonated ester groups were selectively hydrolyzed by the use of TMSBr following the conditions which have been previously reported [39]. Briefly, a round bottom flask was fed with a 5 g of PMAC_2_NP_2_ and 30 mL of anhydrous DCM (30 mL). After the full dissolution of the polymer, TMSBr (7.96 mL, 0.06 mmol) was added dropwise and the solution was left under stirring for 12 h. Finally, MeOH (50 mL) was added and the mixture was stirred for 2 h at ambient temperature. The purified product was obtained after two repeated precipitations in chilled hexane, was dried under vacuum overnight and was characterized by ^31^P NMR spectroscopy, which confirmed the successful cleavage (see Figure S5, Supporting Information). ^31^P NMR (162 MHz, D_2_O) δ: 7.56 (s).

### 2.5. Characterization

#### 2.5.1. Gel Permeation Chromatography (GPC)

The apparent average molecular masses of the synthesized polymers were calculated using a GPC system (Varian 390-LC, Agilent Technologies, Santa Clara, CA, USA) multi-detector equipped with a differential refractive index detector (RI), a light scattering (LS), and a viscosity detector using a guard column (Varian Polymer Laboratories PLGel, Agilent Technologies, Santa Clara, CA, USA) 5 μm, 50 × 7.5 mm) and two ResiPore column’s of the same type. The mobile phase was DMF with 0.1 wt % LiBr adjusted at a flow rate of 1 mL min^−1^ while the columns temperature was thermostated to 70 °C. The GPC system was calibrated using narrow poly(methyl methacrylate) (PMMA) standards ranging from 550 to 1,568,000 g mol^−1^ (EasiVial-Agilent).

#### 2.5.2. Nuclear Magnetic Resonance (NMR) Spectroscopy

^1^H and ^31^P NMR spectra were recorded in CDCl_3_ or D_2_O on a Bruker Avance spectrometer at a proton or phosphorus frequency of 400 MHz and 162 MHz, respectively. Sample temperature was set to 298 K. As external references for spectrometer calibration were TMS for ^1^H NMR and phosphoric acid (H_3_PO_4_) for ^31^P NMR. Chemical shifts are given in parts per million (ppm, [δ]) and were referenced by using the residual signal of the deuterated solvent.

#### 2.5.3. Potentiometric Titration

The titration curves of the pH-responsive polymers in the pH range from 2 to 10 were obtained by monitoring the decrease of the solution pH upon the addition of aliquots of 0.1 M NaOH to a 0.1 wt % polymer solution. The measurements were performed with application of gentle agitation at 25 °C and the pH was monitored using a Thermo Scientific Orion (RL150), (Thermo Fisher Scientific, Waltham, MA, USA) pH-meter. The pH value was measured after the addition of every aliquot only after it remained constant. The dissociation constant *pK*_a_, was calculated from the potentiometric titration curves using the Henderson–Hasselbach equation, at *α* = 0.5, as given by

$$pK_{a}=pH-log\frac{\alpha }{1-\alpha }$$

where *α* is the degree of ionization.

#### 2.5.4. Electrokinetic measurements

Aqueous electrophoretic measurements of the polymer aqueous solutions as a function of pH were conducted using a Malvern Zetasizer NanoZS instrument, (Malvern Instruments, Malvern, Worcestershire, UK). Measurements were carried out in the presence of 0.1 mM KCl background electrolyte. The pH of the polymer solutions was adjusted by adding 0.1 M NaOH or HCl aqueous solutions. Three measurements were recorded for each sample and the zeta potential was calculated from the electrophoretic mobility using the Smoluchowski equation. The electrophoretic mobilities (*μ*) were converted into zeta potentials via the Smoluchowski equation:

$$\zeta =\frac{\mu \times \eta }{\varepsilon }$$

where *η* denotes the viscosity and *ε* the permittivity of the solution.

## 3. Results and Discussion

### 3.1. Synthesis of PMAC_2_NP_2_ Homopolymers via RAFT Polymerization

**Scheme 1:**
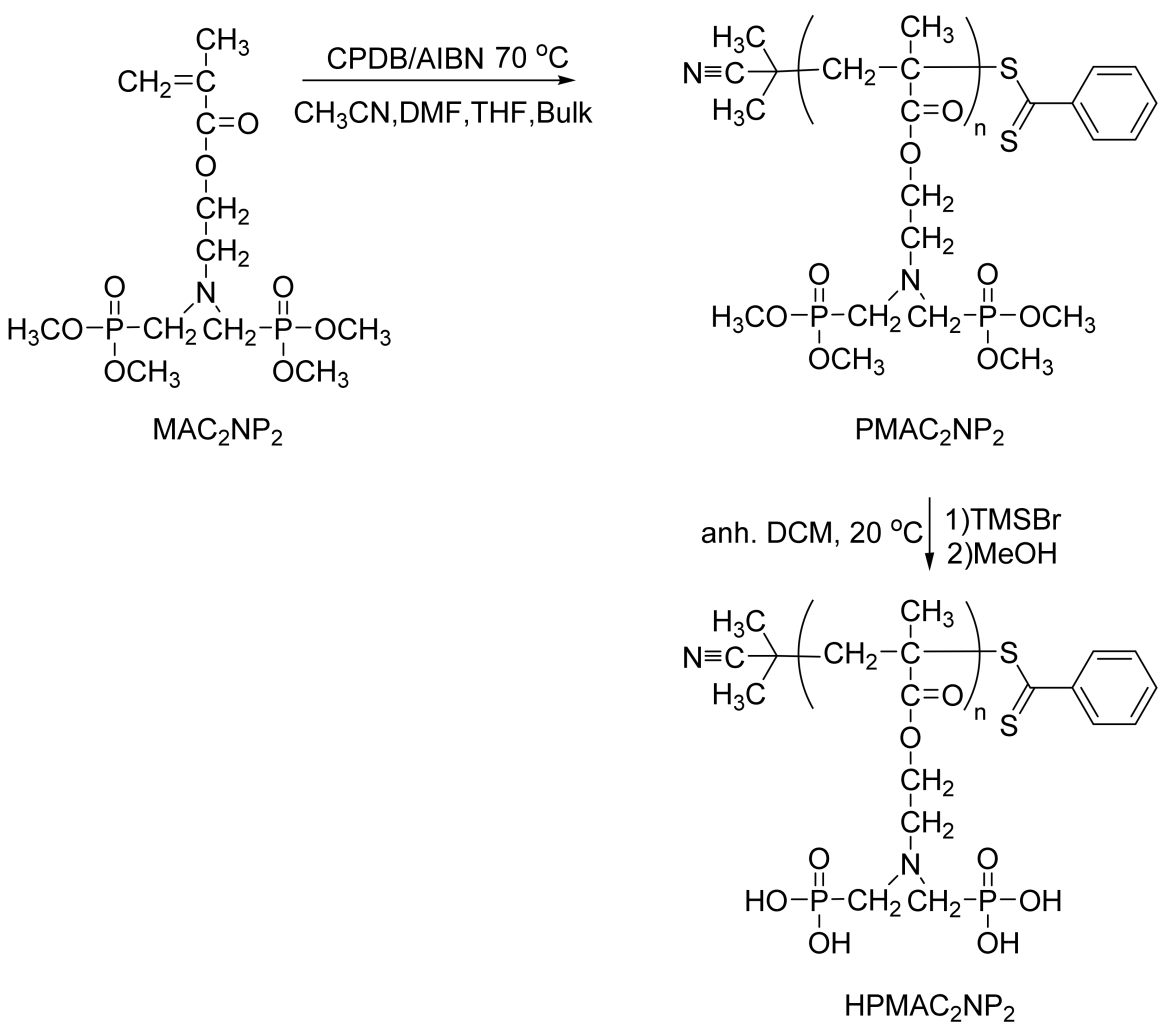
The synthetic pathway for the RAFT polymerization of MAC_2_NP_2_ using CPDB as the transferring agent followed by the hydrolysis of phosphonate into phosphonic acid groups.

The compound 2-cyano-2-propyl-benzodithioate is an efficient controlling agent for RAFT polymerization of methacrylate esters including phosphonate derivatives [25,40,41,42,43]. Therefore, the polymerization of MAC_2_NP_2_ was performed under RAFT conditions using CPDB as the chain transfer agent in the presence of AIBN at a constant molar ratio of CPDB/AIBN = 3 at 70 °C in CH_3_CN maintaining a constant 31/1 MAC_2_NP_2_/CPDB ratio. Similar conditions were used for the polymerization of dimethyl(methacryloyloxy) methyl phosphonate (MAPC1) yielding of well-defined homopolymers with low dispersities [25]. Additionally, in order to assess the influence of the polarity solvent on the kinetics of MAC_2_NP_2_, the polymerizations were also carried out using the aforementioned conditions in less polar solvents including DMF, THF, and in bulk for equal polymerization times (Scheme 1). The polarity of the solvents decreases in the following order CH_3_CN (0.460) > DMF (0.386) > THF (0.207) according to their normalized empirical parameter solvent polarity $E_{N}^{T}$ [44].

The results from the polymerizations in different solvents are summarized in Table 1. The conversion of MAC_2_NP_2_ monomer from the withdrawn samples was calculated from ^1^H NMR by use of the normalized surface area of the reactive double bonds of MAC_2_NP_2_ as compared to the surface area of the methyl protons associated with the phosphonated groups (3.75–3.77 ppm). The number average of molar mass (*M_n_*) and polydispersity indices (*M_w_/M_n_*) were calculated by GPC (using PMMA calibrants) in DMF at 70 °C.

**Figure 1:**
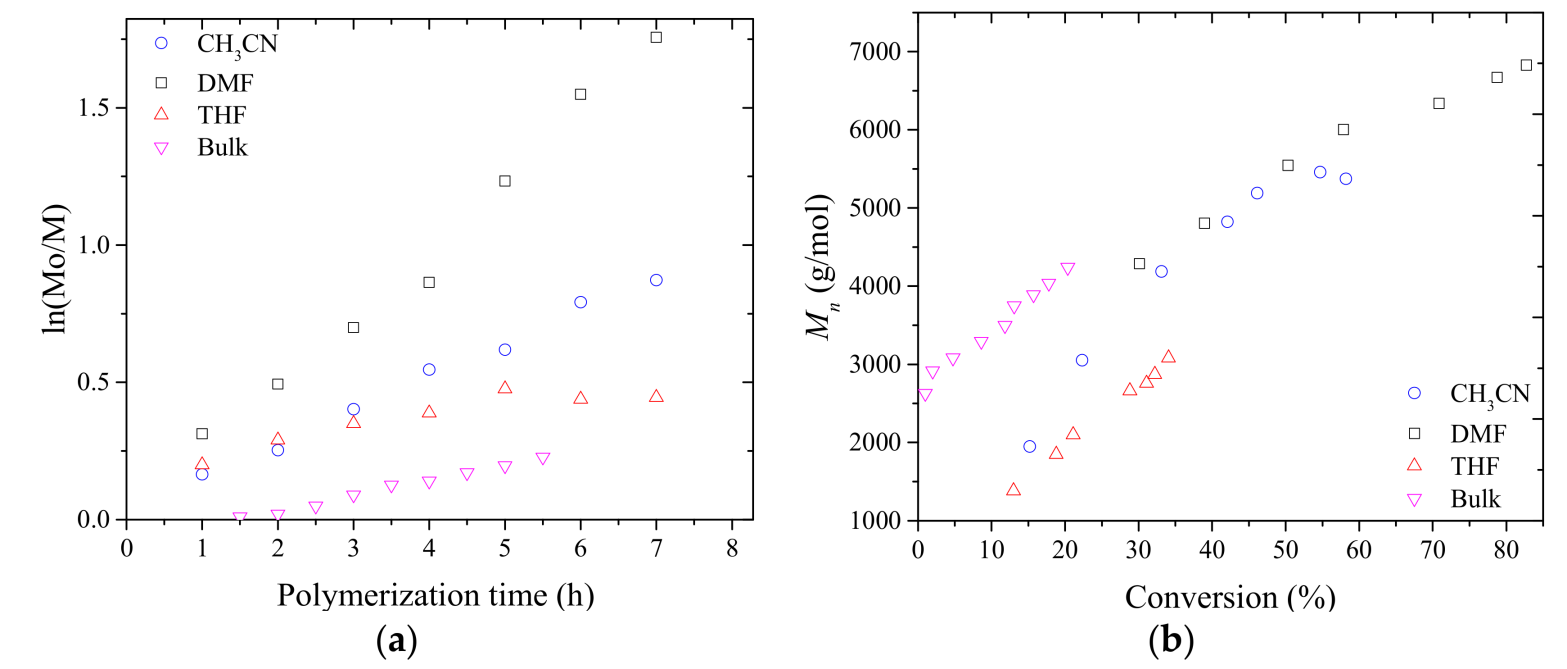

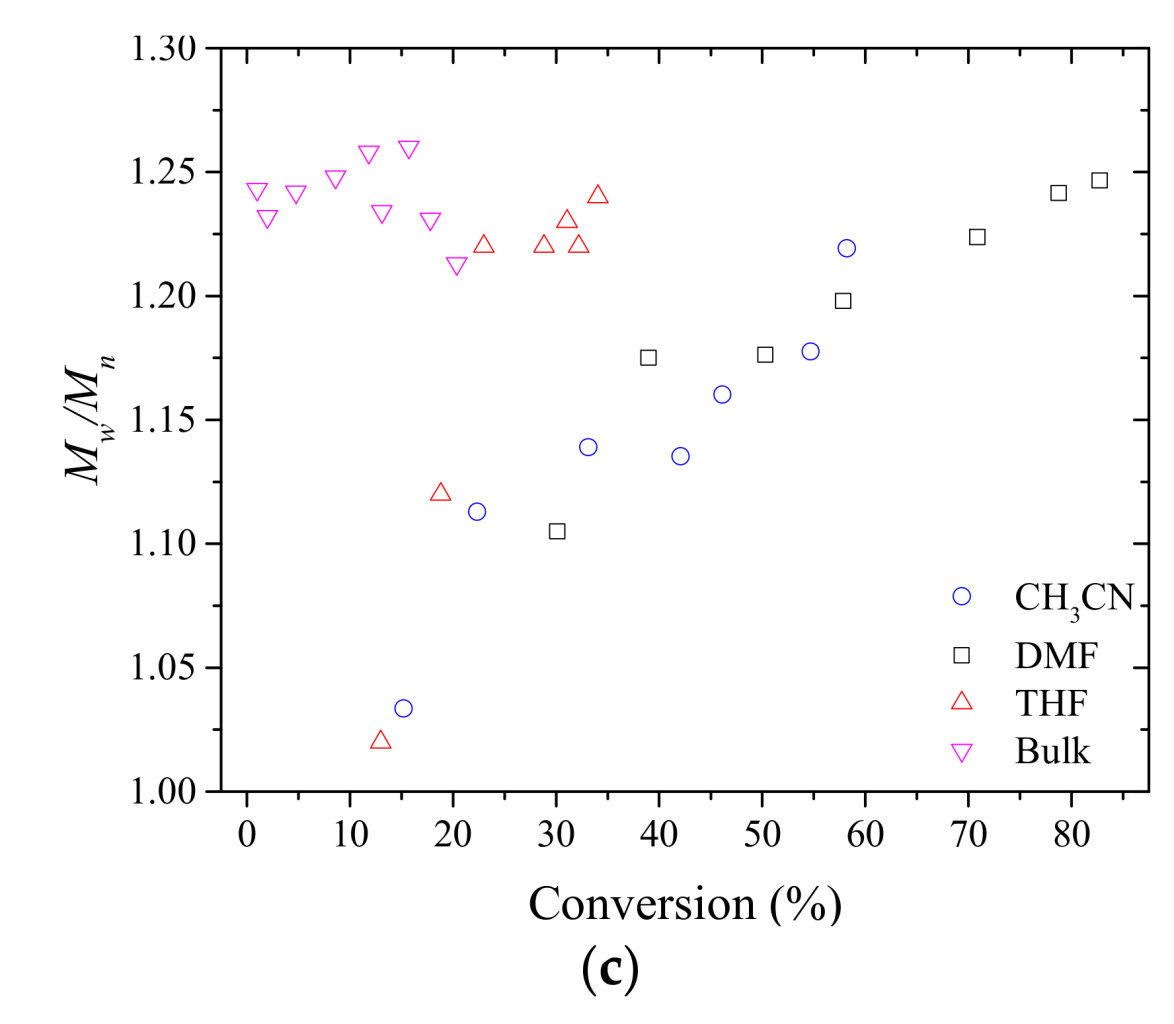
(**a**) The pseudo-first order kinetic plot, (**b**) number average molar mass and (**c**) dispersity index versus conversion plot for the RAFT polymerization of MAC_2_NP_2_ in CH_3_CN, DMF, THF, and bulk at 70 °C.

Figure 1a,b illustrate the pseudo-first-order kinetic plot and the plot of *M_n_*s values as a function of the polymerization time and monomer conversion, respectively for all RAFT polymerizations in different solvents and in bulk. A close inspection of Figure 1a corresponding to the pseudo-first-order plots generated clearly indicates that the polarity of solvents influences the rates of polymerizations. Especially polymerization using DMF as the reaction medium exhibited higher monomer conversions (82%) compared to the other solvents for the same polymerization time (Table 1, Figure 1b). The polymerization did not exhibit any signs of the induction period, reaching 30% monomer conversion within 1 h, but the rate subsequently decelerated. The first-order kinetic plots indicate the presence of a slight deviation of linearity at 60% of conversion, suggesting the loss of the steady conditions. The kinetics plot in CH_3_CN and in bulk presented a linear profile compared to THF in which the first order kinetic plots showed a plateau after 5 h of polymerization indicating that either terminations or transfer to the solvent may occur. The monomer conversion reached 58% and 34% within 7 h for the polymerization in CH_3_CN and THF, respectively. In bulk, the polymerization was retarded due to the extremely high viscosity of the medium, therefore, the conversion reached 20% within 5.5 h and was further increased to 47% until 22 h. At this point, it is noteworthy to mention that the polymerization was performed in a less polar solvent as 1,4-dioxane ($E_{N}^{T}$ = 0.164) under the same conditions, exhibiting lower conversions (17%) after 7 h (data not showed). The number average molar masses evolution with conversion was found to increase in a linear fashion, (Figure 1b) however considerable discrepancy remains between theoretical and experimental molar masses (Table 1). As usual, a wariness should be maintained comparing these data as they are derived from a conventional calibration based on PMMA calibrants and, so, cannot be totally trusted. The evolution of polydispersity indices (*M_w_*/*M_n_*) versus conversion (Figure 1c) exhibited a steady increase as the polymerization proceeding from an initial value of 1.10 to a final value of 1.24 for all the experiments. However, they remained lower than 1.3 verifying the controlled behavior of the RAFT polymerizations.

The results from the above studies suggested that the polarity of the reaction medium has a preeminent impact on the kinetics of the polymerizations. Even though previous reports of RAFT polymerization of MMA using trithiocarbonate chain transfer agents did not show any dependence on solvent polarity [45], in our study, it was noticed that in high polar solvents as CH_3_CN and DMF, the polymerizations present all the characteristics of a controlled process, exhibiting higher rates and higher conversions as well. In the case of bulk, the polymerization also proceeded in a controlled manner, however, it was markedly retarded due to the extremely high viscosity of the medium. One tentative explanation for this behavior might be that MAC_2_NP_2_ is preferentially solvated by polar solvents due its polar nature rendering the propagating radicals less soluble to less polar solvents which, in turn, leads to the retardation or termination of the polymerization as it was noticed when THF or 1,4-dioxane were used as polymerization solvents. Similar results were also noticed for the polymerization of dimethyl(methacryloyloxy) methyl phosphonate (MAPC1) in solvents with different polarities [25].

### 3.2. Hydrolysis of PMAC_2_NP_2_ Polymers

The homopolymer of PMAC_2_NP_2_ synthesized in DMF was chosen for hydrolysis of the phosphonated ester groups by the use of TMSBr and the subsequent addition of methanol, which is considered a very effective method for the cleavage of phosphonate ester groups without affecting the chemical structure of the polymer. The successful hydrolysis was confirmed via ^31^P NMR (see Figure S5 Supporting information) in D_2_O where the shifting of the phosphorus peak from 26.55 to 7.56 ppm constitutes concrete evidence for the total hydrolysis of the phosphonate ester groups. The hydrolyzed polymer which will be referred from now on as HPMAC_2_NP_2_.

### 3.3. Aqueous Solution Properties of HPMAC_2_NP_2_ Homopolymer

**Scheme 2:**
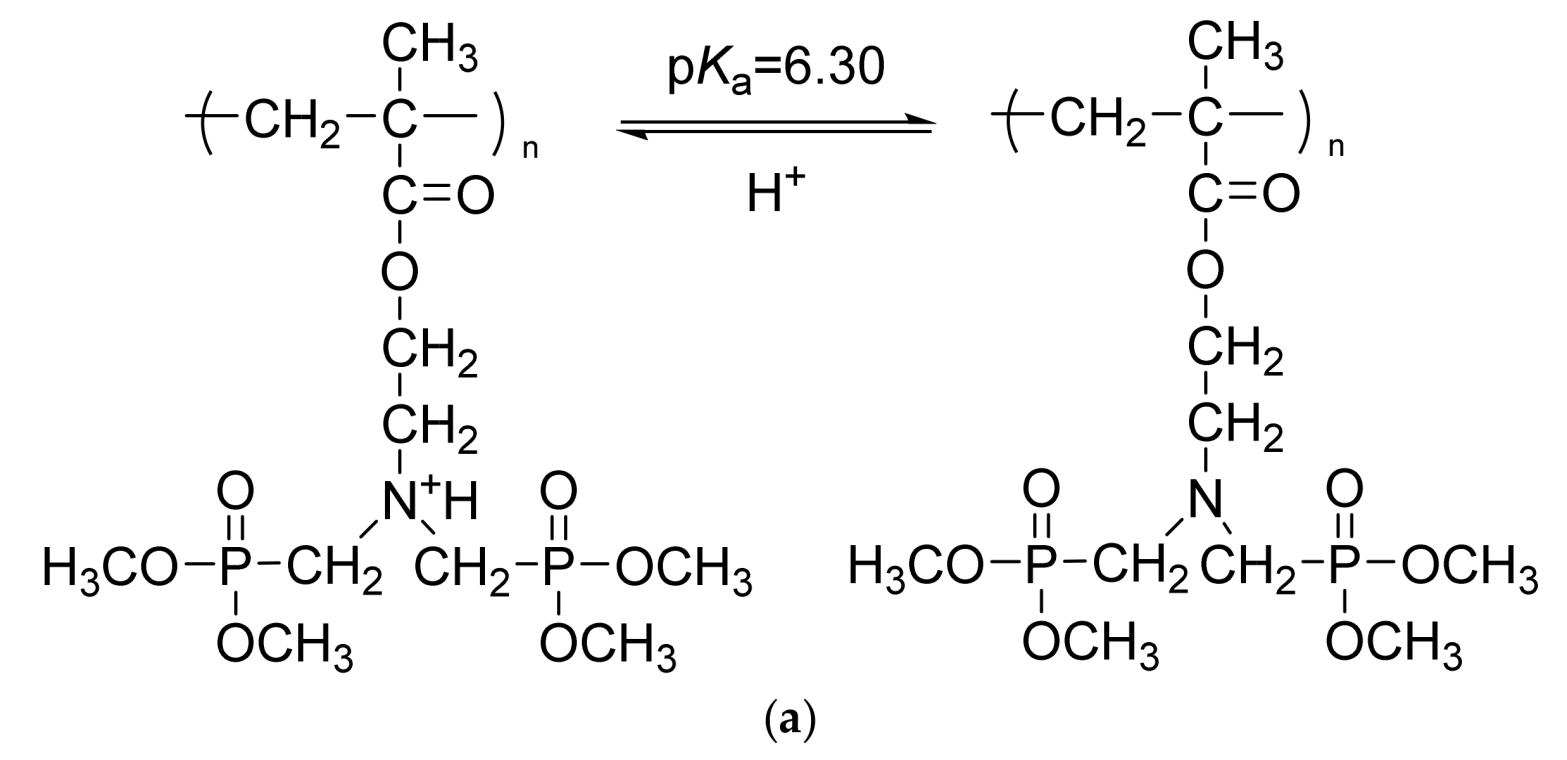

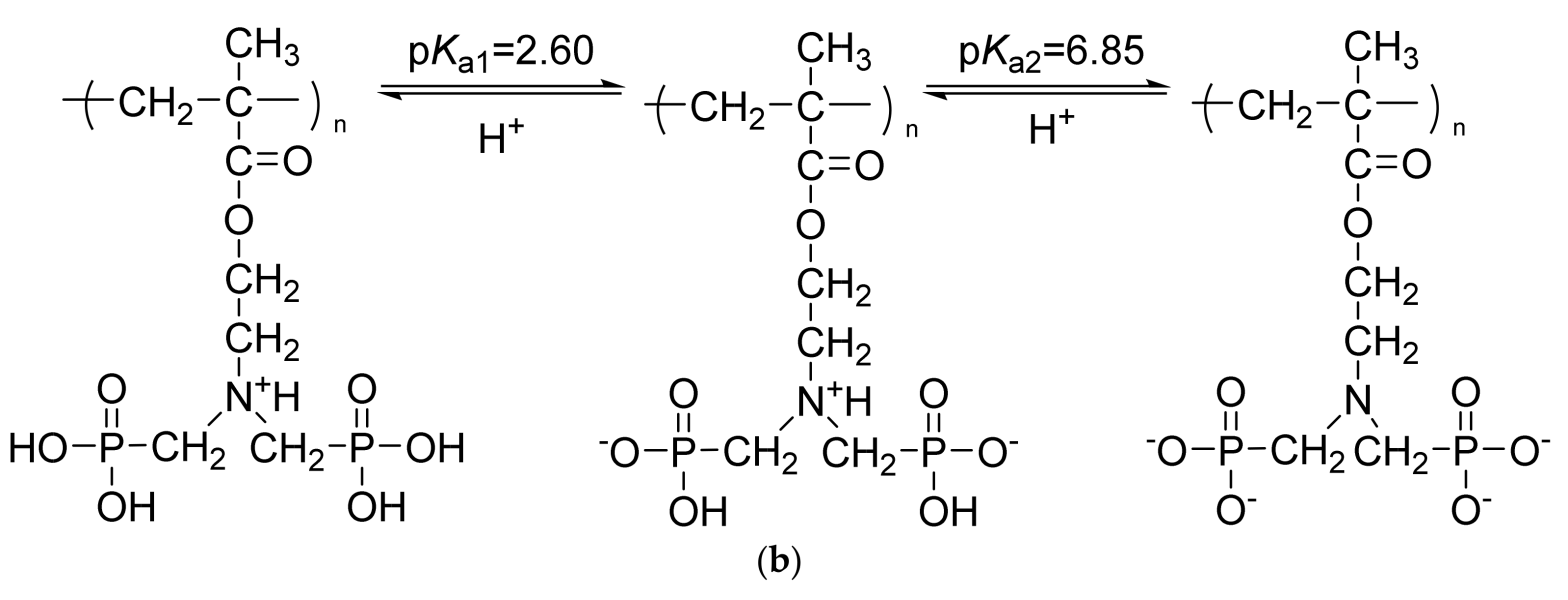
(**a**) The reversible protonation process of PMAC_2_NP_2_ and (**b**) HPMAC_2_NP_2_.

MAC_2_NP_2_ is an ionizable monomer possessing a tertiary amine unit, which behaves as a weak base and participates in a weak acid-base equilibrium upon changing the solution pH exhibiting one dissociation constant *pK*_a_. The reversible protonation process of the PMAC_2_NP_2_ homopolymer is illustrated in Scheme 2a. Hydrolysis of the phosphonate ester groups leads to the formation of two pairs of phosphonic acid groups which behave as a weak diacid presenting two dissociation equilibria with dissociation equilibrium constants of *pK*_a1_ and *pK*_a2_ where, in the case of phosphonic acid, the *pK*_a1_ ranges from 2 to 3 and the *pK*_a2_ from about 6–7 (Scheme 2b) [25,46].

**Figure 2:**
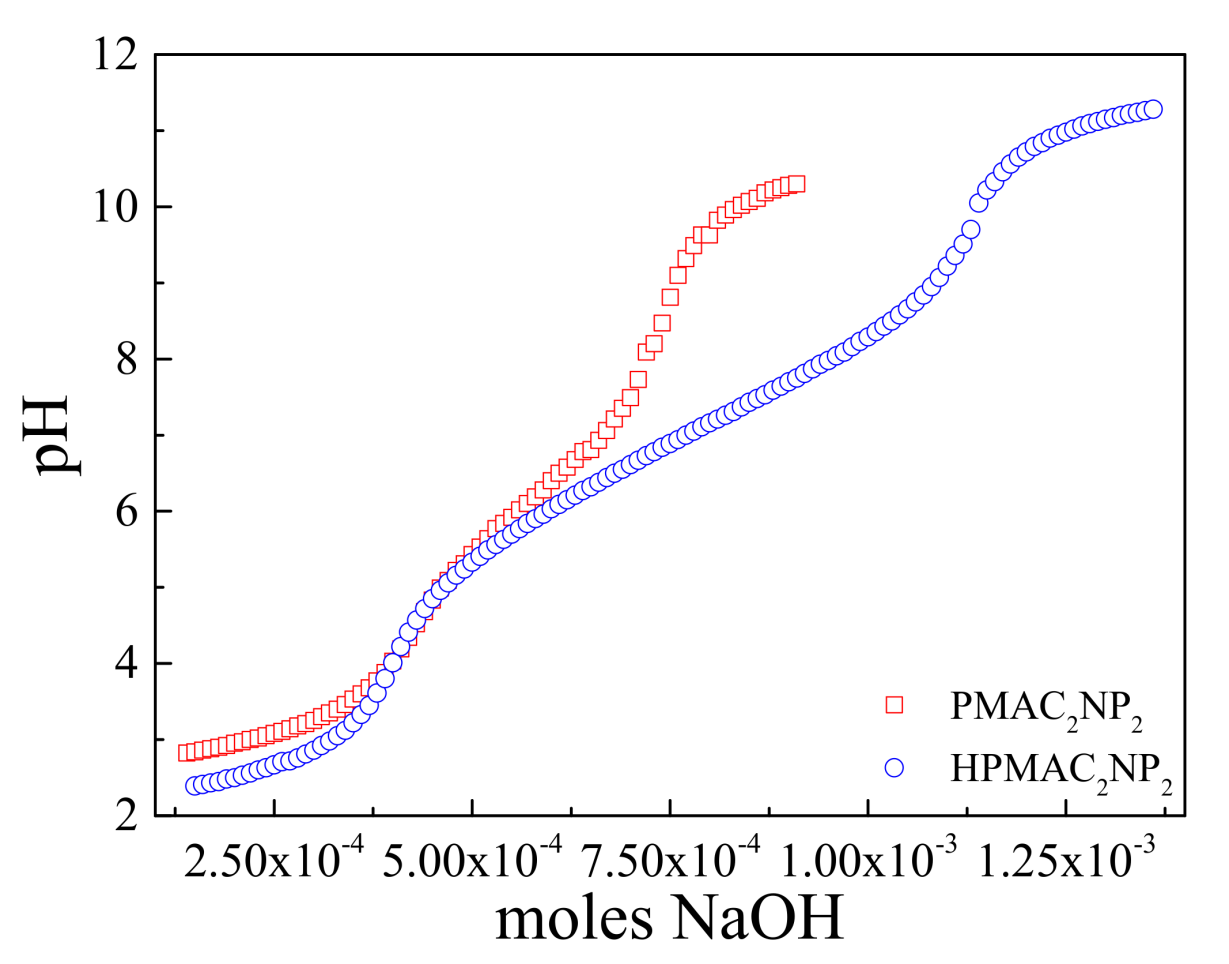
The titration curves for the PMAC_2_NP_2_ (□) and HPMAC_2_NP_2_ (○) polymers at *c* = 0.1 wt %.

The effect of the solution pH at the ionization behavior of PMAC_2_NP_2_ polymers before and after hydrolysis of the phosphonate groups was investigated by potentiometric titration and zeta potential measurements. The titration curve of PMAC_2_NP_2_ is presented in Figure 2. The curve exhibited a single ionization plateau in the pH range from 4 to 8, which is attributed to the deprotonation of the tertiary amines. In particular, at pH around 4.5, the addition of base caused a steep increase of the pH to a value of 5. At this point, further addition of the base results in the deprotonation of the tertiary amine groups of the polymers and leads to a slight increase of the pH (plateau region). Finally, when the basic units have become fully deprotonated, an abrupt increase in the solution pH is observed (pH 8.5) due to the excess base added in solution. The effective *pK*_a1_ was calculated quite accurately from the plot of the pH versus the degree of ionization (α) for α = 0.5 and was found to be 6.30.

For comparison, the titration curve of HPMAC_2_NP_2_ is displayed as well. The latter exhibited a single ionization plateau and well-defined inflection points at pH 4.0 and 9.8, signifying unambiguously the simultaneous deprotonation (from acidic to basic pH) of the ionizable tertiary amine and phosphonic acid groups due to similar buffering zones of the respective units. Especially in the solution of pH 2, the phosphonic acid groups are unionized and the amino groups are totally protonated. An increase of the solution pH initially leads to deprotonation of the first pair of phosphonic acid groups where its completion is noticed at the first inflection point at pH 4. Subsequently, the gradual deprotonation of the amine units takes place with a concomitant ionization of the second pair of phosphonic acid groups. This parallel procedure also rationalizes the extension of the plateau of the titration curve compared to PMAC_2_NP_2_. Finally, when the pH is above 10, the amine groups are considered totally deprotonated while the phosphonic acid groups exist in their ionized (dibasic) form. Moreover, the question arises how the *pK*_a_ value is influenced by the presence of the phosphonic acids, thus, the apparent *pK*_a1_ and *pK*_a2_ which correspond to the primary and secondary dissociation where calculated and found to be 2.60 and 6.85, respectively. Previous studies on the dissociation behavior of methacrylate bearing phosphonic acid as the pendant groups have reported the dissociation constants at *pK*_a1_ and *pK*_a2_ to be 2.60 and 8.20, respectively [25]. In our case, the *pK*_a2_ was significantly shifted to a lower pH which is attributed to the interaction of the amine with the phosphonic acid groups which, in turn, tends to lower the *pK*_a2_ of the latter [47].

**Figure 3:**
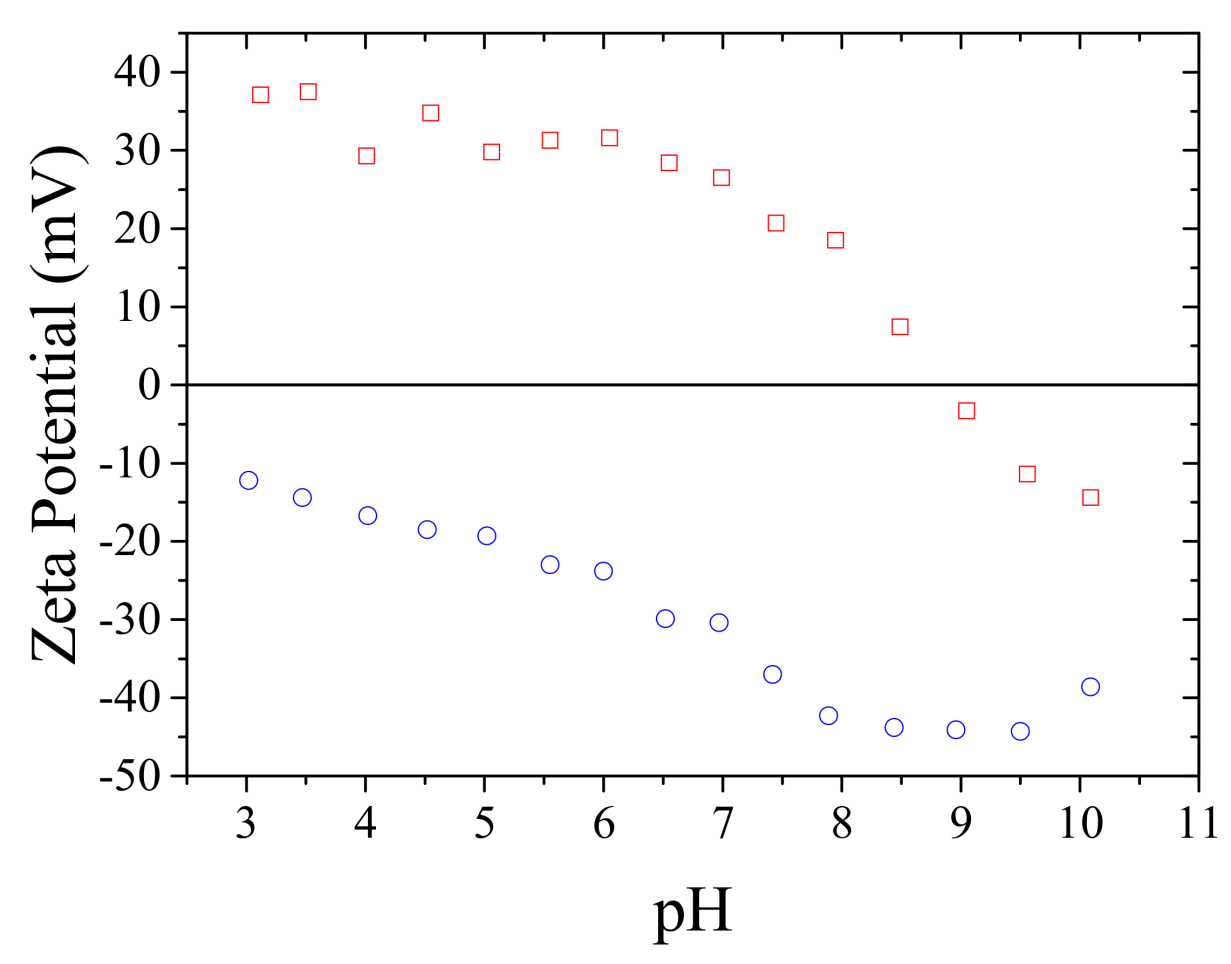
The zeta potential of the PMAC_2_NP_2_ (□) and HPMAC_2_NP_2_ (○) polymers as a function of the solution pH.

In order to gain further insight into the aqueous solution properties of the pH-responsive polymers, data for the zeta potential as a function of pH were also recorded. Figure 3 illustrates the zeta values of PMAC_2_NP_2_ and HPMAC_2_NP_2_ polymers as a function of solution pH. The zeta potential-pH profiles displayed opposite trends for each type of polymer, as was expected. For PMAC_2_NP_2_ starting under acidic conditions, constant positive zeta potential values were reported due to the positive charge of the tertiary amine groups. As the pH further increases, the zeta potential steadily drops due to the charge neutralization of the amine groups and reaches the isoelectric point at pH 8.6 (which is in accordance with the second inflection point (pH = 8.5) of the titration curve of PMAC_2_NP_2_), indicating the zero net charge for the polymers. The zeta potential profile of the HPMAC_2_NP_2_ polymers reverses markedly from positive to negative values for the same pH range examined. Starting from basic conditions, negative zeta values are reported which is attributed to the presence of both pairs of negatively charged phosphonic acid groups. As the solution pH decreases, the zeta potential remains approximately constant until pH 8 where it constantly increases up to pH 3, signifying the concomitant protonation of the second pair of phosphonic acid as well as the amino moieties. The zeta potential values remain negative at this pH regime due to the excess of the negatively charged phosphonic acid groups compared to the positively charged amine groups.

## 4. Conclusions

In the present contribution, we reported for the first time a study on the RAFT polymerization and the aqueous solution properties of an aminobisphosphonated methacrylate. In this preliminary study, an interesting kinetic feature was observed regarding the effect of the polarity of the solvents on polymerization kinetics of MAC_2_NP_2_. Relatively controlled polymerization with controlled number average molar masses and low dispersities showing high conversions and fast polymerization rate were accomplished when using CH_3_CN or DMF as the reaction medium. Decreasing the polarity of the solvent or in bulk conditions led either to the termination or to the retardation of the polymerizations. This kind of behavior is on account of the polar nature of the MAC_2_NP_2_ monomer which is difficult to solubilize in less polar solvents. Hydrolysis of the phosphonate ester groups led to the formation of the phosphonic acid form polymer which exhibited a strong dependence of the solution pH. Aqueous solution characterization of the HPMAC_2_NP_2_ was carried out using potentiometric titration and zeta potential measurements. Potentiometric titrations of the hydrolyzed polymers showed an enhanced plateau regime where the deprotonation of the acidic and basic moieties took place concurrently. Measurements of the zeta potential as a function of pH of PMAC_2_NP_2_ and HPMAC_2_NP_2_ demonstrated opposite profiles due to the presence of both ionizable groups which had a reflection at the zeta potential values. A more detailed study in RAFT polymerization of MAC_2_NP_2_ is on progress in order to explore the impact of temperature of MAC_2_NP_2_ in DMF and, additionally, to examine the association behavior of the hydrolyzed form as a function of pH and salt concentration and the results will be described in forthcoming publications.

## Figures and Tables

**Table 1:** The molecular characteristics of the mediated RAFT polymerization of PMAC_2_NP_2_ in different solvents at 70 °C.

Entry	Solvent	Conv. (%) ^a^	*M_n_* ^b^ (g/mol)	*M_n,th._* ^c^(g/mol)	*M_w_/M_n_*
1	DMF	82	6800	9500	1.24
2	CH_3_CN	58	5300	6700	1.21
3	THF	34	3000	4800	1.14
4	Bulk	47 ^d^	4600	5400	1.21

^a^ Conversion of double bonds as determined by ^1^H NMR at 7 h of polymerization, ^b^ Determined by PMMA calibrated GPC, ^c^ *M_n_,_th._* = ([MAC_2_NP_2_]_0_/[CPDB]_0_ × MW_MAC2NP2_ × conv. + MW_CPDB_), ^d^ Conversion calculated at 22 h of polymerization.

## Supplementary Materials

The following are available online at http://www.mdpi.com/2073-4360/10/7/711/s1.
