# Supplementary material for: Synthesis and Aqueous Solution Properties of an Amino Bisphosphonate Methacrylate Homopolymer via RAFT Polymerization
Source: Polymers (Basel). 2018 Jun 27;10(7):711. doi: 10.3390/polym10070711 (PMC6404032; doi:10.3390/polym10070711)
Supplement: Supplementary file 1 [file polymers-10-00711-s001.pdf]

Supporting Information

Synthesis and Aqueous Solution Properties of an Amino Bisphosphonate Methacrylate Homopolymer via RAFT Polymerization

Panagiotis G. Falireas, Claire Negrell, Ghislain David*

Institut Charles Gerhardt–UMR-CNRS 5253, Ingénierie et Architectures Macromoléculaires, ENSCM, 8 rue de l'Ecole normale, 34296 Montpellier Cedex 5, France

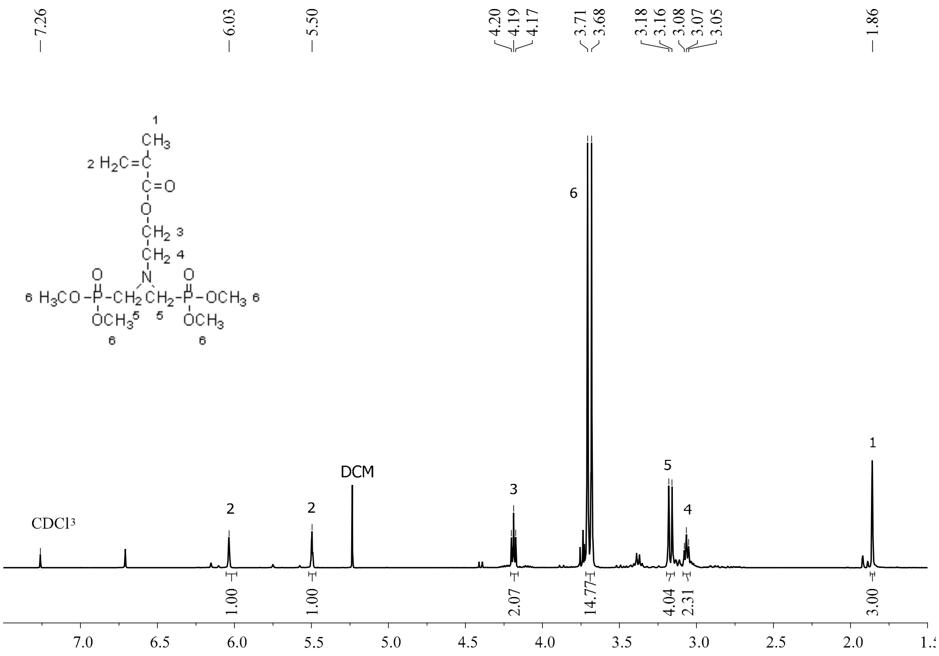

Figure S1. ^{1}H NMR spectrum (in $CDCl_3$) for the MAC_2NP_2.

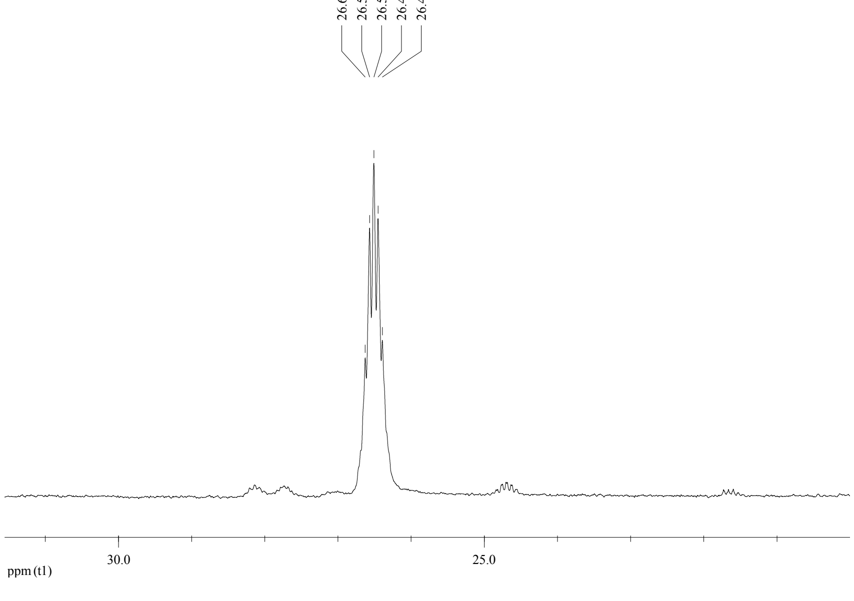

Figure S2. ^{31}P NMR spectrum (in $CDCl_3$) for the MAC_2NP_2.

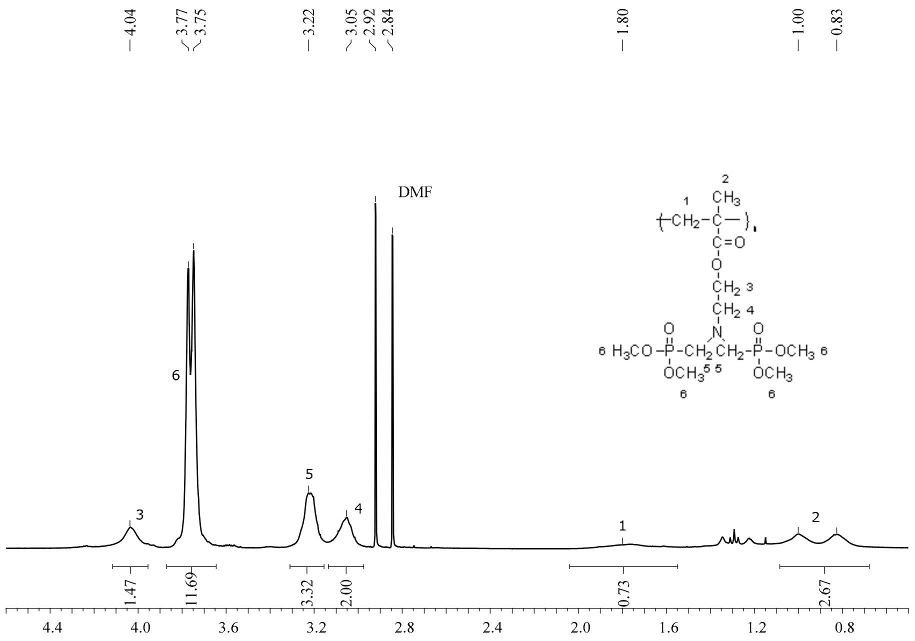

Figure S3 ^{1}H NMR spectrum (in $CDCl_3$) for the $PMAC_2NP_2$ synthesized via RAFT in DMF.

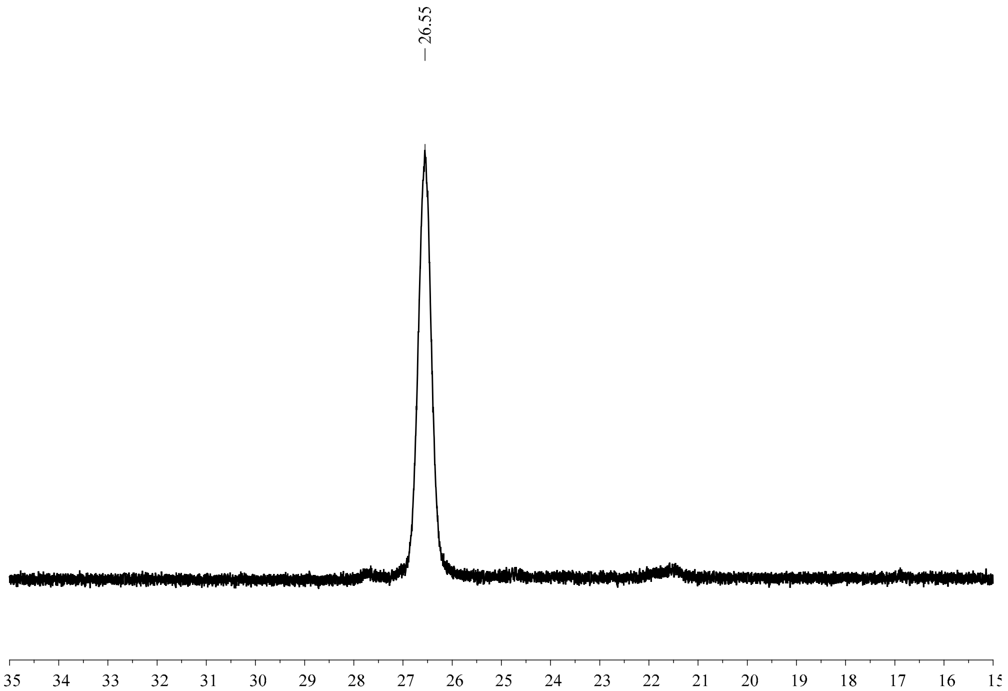

Figure S4. ^{31}P NMR spectrum (in $CDCl_3$) for the $PMAC_2NP_2$ synthesized via RAFT in DMF.

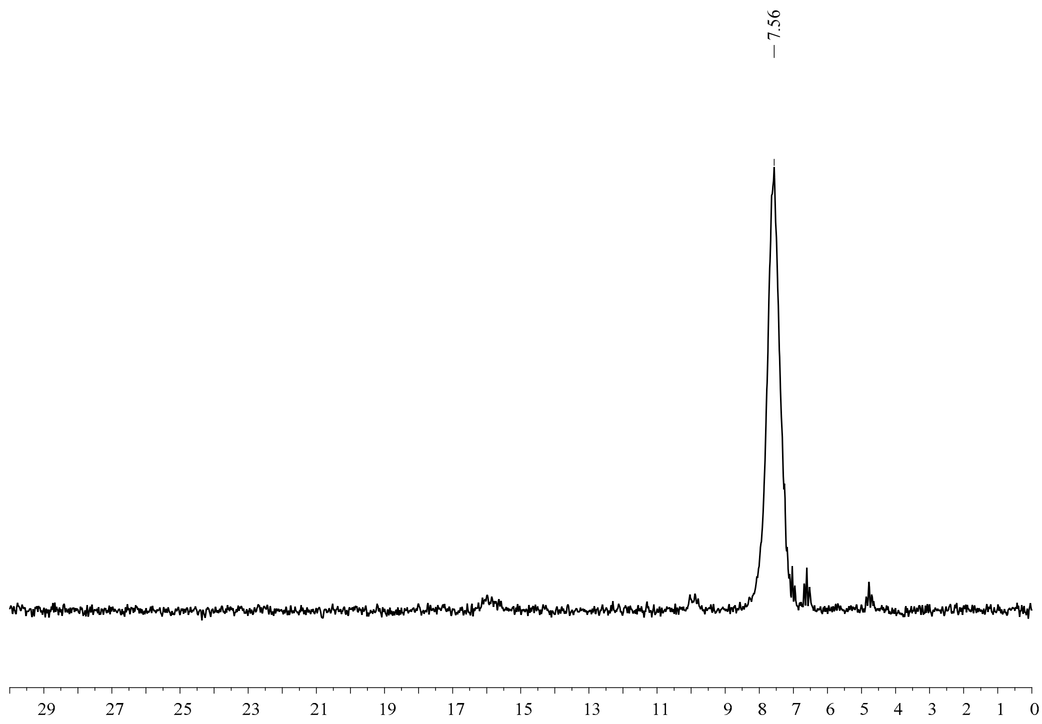

Figure S5. ^{31}P NMR spectrum (in D_2O) for the $HPMAC_2NP_2$ synthesized via RAFT in DMF after hydrolysis.
